# Supplementary material for: Living on the edge: urban fireflies (Coleoptera, Lampyridae) in Morelia, Michoacán, Mexico
Source: PeerJ. 2023 Dec 14;11:e16622. doi: 10.7717/peerj.16622 (PMC10725667; doi:10.7717/peerj.16622)
Supplement: Supplemental Information 1 [file peerj-11-16622-s001.docx]

**SUPPLEMENTARY MATERIAL**

**Table S1.** Sites from Morelia city and surrounding areas where firefly populations have been reported in the last decades.

| **Urban zone** | **Firefly population status** | **Year of observation** | **Latitude (°N)** | **Longitude (°W)** | **Elevation (m asl)** | **Vegetated spaces category** | **Terrain slope** | **Associated waterbodies** | **Source** |
| --- | --- | --- | --- | --- | --- | --- | --- | --- | --- |
| Extra-urban | Extant | 2021 | 19.5397 | -101.2393 | 2213 | Meadows | Steep slope | None | Naturalista, 2021 |
| Extra-urban | Extant | 2022 | 19.5397 | -101.2485 | 2153 | Woods | Steep slope | Permanent river | Entomological sampling 2021-2022 |
| Extra-urban | Extant | 2021 | 19.5739 | -101.1237 | 2362 | Hills | Steep slope | Temporal river | Naturalista, 2021 |
| Extra-urban | Extant | 2021 | 19.6012 | -101.3489 | 2384 | Cultivated land | Hillside | None | Naturalista, 2021 |
| Extra-urban | Extant | 2020 | 19.6427 | -101.1377 | 2108 | Hills | Foothill | Temporal river | Naturalista, 2021 |
| Extra-urban | Extant | 2021 | 19.6651 | -101.1414 | 2123 | Hills | Foothill | Permanent river | Naturalista, 2021 |
| Extra-urban | Possible extant | 1963 | 19.6833 | -101.1167 | 1962 | Hills | Hillside | None | GBIF, 2022 |
| Peri-urban | Extant | 2022 | 19.6248 | -101.2447 | 2013 | Woods | Hillside | Permanent lake | Entomological sampling 2021-2022 |
| Peri-urban | Extant | 2022 | 19.6339 | -101.2866 | 1999 | Meadows | Flat plains | Permanent creek | Entomological sampling 2021-2022 |
| Peri-urban | Extant | 2022 | 19.6428 | -101.2662 | 1898 | Wetland | Flat plains | Permanent wetland | Entomological sampling 2021-2022 |
| Peri-urban | Extant | 2020 | 19.6434 | -101.2182 | 2024 | Residential area | Foothill | Temporal river | Naturalista, 2021 |
| Peri-urban | Extant | 2021 | 19.6447 | -101.274 | 1889 | Wetland | Flat plains | Permanent wetland | Naturalista, 2021 |
| Peri-urban | Extant | 2022 | 19.6479 | -101.224 | 1983 | Meadows | Flat plains | Permanent lake | Entomological sampling 2021-2022 |
| Peri-urban | Extant | 2022 | 19.6483 | -101.1983 | 2045 | Meadows | Foothill | Temporal creek | Entomological sampling 2021-2022 |
| Peri-urban | Extant | 2020 | 19.6490 | -101.1575 | 2113 | Cultivated land | Flat plains | None | Naturalista, 2021 |
| Peri-urban | Extant | 2016 | 19.6505 | -101.2278 | 1971 | Roads | Flat plains | Permanent lake | Naturalista, 2021 |
| Peri-urban | Extant | 2019 | 19.6506 | -101.2226 | 1963 | Public garden | Flat plains | Permanent lake | Naturalista, 2021 |
| Peri-urban | Extant | 2019 | 19.6629 | -101.2258 | 1914 | Vacant lots | Flat plains | Temporal river | Naturalista, 2021 |
| Peri-urban | Extant | 2022 | 19.6645 | -101.2109 | 1930 | Meadows | Flat plains | Permanent channel | Entomological sampling 2021-2022 |
| Peri-urban | Extant | 2021 | 19.6663 | -101.2082 | 1981 | Roads | Flat plains | Permanent channel | Naturalista, 2021 |
| Peri-urban | Extant | 2021 | 19.6829 | -101.2474 | 1891 | Vacant lots | Flat plains | Permanent channel | Naturalista, 2021 |
| Peri-urban | Extant | 2022 | 19.6853 | -101.3168 | 1980 | Public garden | Flat plains | None | Entomological sampling 2021-2022 |
| Peri-urban | Possible extant | 1972 | 19.6883 | -101.3047 | 1962 | Vacant lots | Flat plains | Permanent pond | GBIF, 2022 |
| Peri-urban | Possible extant | 1988 | 19.6997 | -101.1167 | 2051 | Vacant lots | Foothill | Temporal creek | Naturalista, 2021 |
| Peri-urban; extinction zone | Extinct | 1950s | 19.7433 | -101.2073 | 1904 | Public garden | Flat plains | None | Interviews, 2022 |
| Urban core | Extant | 2018 | 19.6731 | -101.2001 | 1966 | Backyard | Hillside | Permanent river | Naturalista, 2021 |
| Urban core | Extant | 2021 | 19.674 | -101.211 | 1937 | Vacant lots | Flat plains | None | Entomological sampling 2021-2022 |
| Urban core | Extant | 2021 | 19.6764 | -101.2057 | 1944 | Cultivated land | Flat plains | Permanent river | Entomological sampling 2021-2022 |
| Urban core | Extant | 2022 | 19.6826 | -101.2066 | 1904 | Public garden | Foothill | Permanent channel | Entomological sampling 2021-2022 |
| Urban core | Extant | 2022 | 19.6893 | -101.2499 | 1898 | Meadows | Flat plains | Permanent channel | Entomological sampling 2021-2022 |
| Urban core | Extant | 2022 | 19.6926 | -101.208 | 1921 | Public garden | Flat plains | None | Entomological sampling 2021-2022 |
| Urban core | Extant | 2019 | 19.6964 | -101.2333 | 1886 | Meadows | Flat plains | Permanent wetland and channels | Naturalista, 2021 |
| Urban core | Extant | 2022 | 19.6964 | -101.2349 | 1898 | Wetland | Flat plains | Permanent wetland | Entomological sampling 2021-2022 |
| Urban core; extinction zone | Extinct | 1980s | 19.6683 | -101.2262 | 1889 | Residential area | Flat plains | None | Interviews, 2022 |
| Urban core; extinction zone | Extinct | 1980s | 19.6858 | -101.1995 | 1906 | Residential area | Flat plains | None | Interviews, 2022 |
| Urban core; extinction zone | Extinct | 1950s | 19.6917 | -101.1855 | 1907 | Residential area | Flat plains | Permanent river | Interviews, 2022 |
| Urban core; extinction zone | Extinct | 1980s | 19.6933 | -101.2054 | 1908 | Residential area | Flat plains | None | Interviews, 2022 |

**Table S2. Variables and categories were evaluated to determine the current habitat of the firefly populations from Morelia city.**

1. Geographic location and elevation
2. Habitat suitability variables
   1. Landscape class: urban land use classification in 2021, based on MacGregor-Fors (2011):
      1. Commercial
      2. Conservation
      3. green area
      4. residential
      5. industrial
   2. Type of vegetated space: forests or woods, shrubs, meadows, cultivated land, green urban spaces (e.g., backyards or domestic gardens, parks, open grass, sport areas, public gardens), vacant lots, corridors (e.g., median strips, along rivers, or between residential areas), conservation areas, hills.
   3. Habitat size: limited to the area within a 500-m radius circular buffer (0.79 km^2^)
   4. Percentage of suitable habitat: estimated green area within a ratio of 500-m radius circular buffer (0.79 km^2^).
      - 1. highly vegetated (67–100% vegetated area)
        2. moderately vegetated (34–66% vegetated area)
        3. sparsely vegetated (0–33% vegetated area)
   5. Watercourses:
      1. Type: temporary or permanent lake, creek, river, pond, channel, and wetland.
      2. Distance from firefly population (m)
      3. Size: width
   6. Terrain slope (FAO categorization):
      1. very gentle slopes (0-4% slope gradient)
      2. gentle slopes (>4-8%)
      3. moderate slopes (>8-16%)
      4. steep to extremely steep slopes (>16%)
3. Disturbance factors
   1. Urbanization degree (following MacGregor-Fors 2010, 2011)
      1. Urban-core or intra-urban spaces
      2. Peri-urban
      3. Extra-urban
      4. Extinction zone
   2. Percentage of urban surfaces within a ratio of 500-m radius circular buffer (0.79 km^2^)
      1. sparsely developed (0–33% built cover)
      2. moderately developed (34–66% built cover)
      3. highly developed (67–100% built cover)
   3. Radiance values. Based on the database of Earth Observation Group (2021).

**Table S3. Updated species list of fireflies (Coleoptera, Lampyridae) for Michoacán, México.** Based on Pérez-Hernández et al. (2022), Zaragoza-Caballero et al. (2023), and this study. ** New records for Michoacán.

| **Lampyridae from Michoacán** |
| --- |
| *Aspisoma aegrota* (Gorham, 1880) |
| *Aspisoma depictum* (Gorham, 1880) |
| *Aspisomoides bilineatum* (Gorham, 1880) |
| *Bicellonycha amoena* (Gorham, 1880) |
| *Cratomorphus halffteri* Zaragoza-Caballero, 2012** |
| *Photinus acutiformis* Zaragoza-Caballero & Cifuentes-Ruíz, 2023** |
| *Photinus advenus* Olivier, 1907 |
| *Photinus aliciae* Zaragoza-Caballero, 2000 |
| *Photinus aliciarodriguezae* Zaragoza-Caballero & López-Pérez, 2023 |
| *Photinus anisodrilus* Zaragoza-Caballero, 2007** |
| *Photinus apahtzii* Zaragoza-Caballero & López-Pérez, 2023 |
| *Photinus ater* Gorham, 1881 |
| *Photinus barrerae* Zaragoza-Caballero & Rodríguez-Mirón, 2023 |
| *Photinus brailovskyi* Zaragoza-Caballero, 2017 |
| *Photinus chanantzkua* Zaragoza-Caballero & Domínguez-León, 2023 |
| *Photinus chipirietetsi* Zaragoza-Caballero & Vega-Badillo, 2023** |
| *Photinus extensus* Gorham, 1881** |
| *Photinus guillermodeltoroi* Zaragoza-Caballero & Rodríguez-Mirón, 2023** |
| *Photinus intermedius* Zaragoza-Caballero, 1995 |
| *Photinus leobonillai* Zaragoza-Caballero & Domínguez-León 2023 |
| *Photinus marthae* Zaragoza-Caballero & Cifuentes-Ruiz, 2023 |
| *Photinus ortegae* Zaragoza-Caballero & Rodríguez-Mirón, 2023 |
| *Photinus parvusater* Zaragoza-Caballero, 1995 |
| *Photinus* ca. *pyralis* (Linnaeus, 1767) (under *P. pyralis* in Zaragoza-Caballero et al. 2023) |
| *Photinus vegai* Zaragoza-Caballero & Cifuentes-Ruíz, 2020** |
| *Photinus vulgatus* Olivier, 1907 |
| *Photinus zuritai* Zaragoza-Caballero & Cifuentes-Ruiz, 2023** |
| *Photinus* ca. *brailovskyi* Zaragoza-Caballero, 2017 |
| *Photinus* sp. 2 ** |
| *Photinus* sp. 3** |
| *Photuris fulvipes (*Blanchard, 1846)** |
| *Photuris lugubris* Gorham, 1881** |
| *Photuris* group *versicolor* (Fabricius, 1798) |
| *Photuris* sp. |
| *Pleotomus emmiltos* Zaragoza-Caballero, 2002** |
| *Pleotomus pallens* LeConte, 1866 |
| *Pyractomena striatella* Gorham, 1880** |
| *Pyractomena* sp. |
| *Pyropyga minuta* (LeConte, 1851) |
| *Pyropyga alticola* Green, 1961** |
| *Pyropyga nigricans* (Say, 1823) |

**
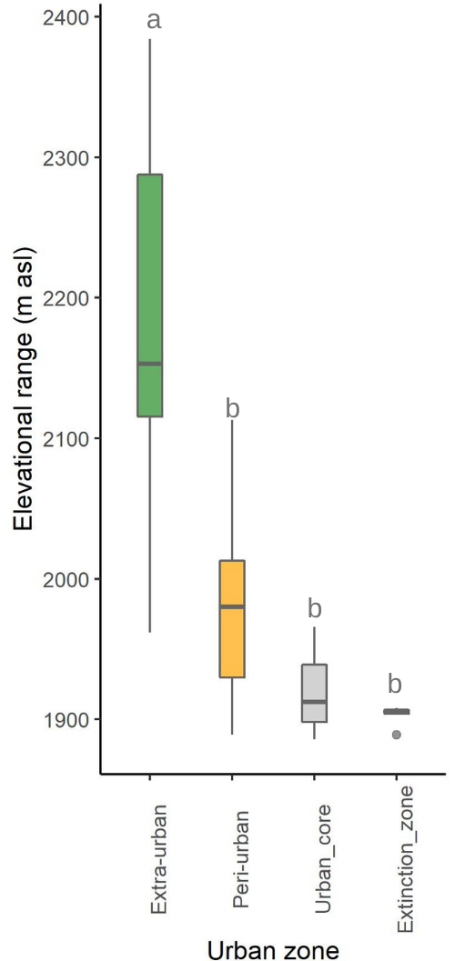
**

**Figure S1.** Elevational range among sites where firefly populations have been recorded from different zones in Morelia city, Michoacán, Mexico.
